# Immunological Differences in Human Peripheral Blood Mononuclear Cells Treated with Traditional Japanese Herbal Medicines Hochuekkito, Juzentaihoto, and Ninjin'yoeito from Different Pharmaceutical Companies

**DOI:** 10.1155/2021/7605057

**Published:** 2021-09-18

**Authors:** Anna Kiyomi, Ayaka Matsuda, Moeko Nara, Kyosuke Yamazaki, Shinobu Imai, Munetoshi Sugiura

**Affiliations:** Department of Drug Safety and Risk Management, School of Pharmacy, Tokyo University of Pharmacy and Life Sciences, Hachioji, Tokyo 192-0392, Japan

## Abstract

Hochuekkito (HET), Juzentaihoto (JTT), and Ninjin'yoeito (NYT) have been used as Hozai, a group of traditional Japanese herbal medicines, to treat physically and mentally weak cancer patients. Their compositions are quite different, and Japanese pharmaceutical companies have been using different types or quantities of herbs for formulations with the same name. Here, we compared the immunological differences between HET, JTT, and NYT with respect to the induced T cell subsets and cytokines. Peripheral blood mononuclear cells (PBMCs) were isolated from healthy volunteers and treated with 0 (control), 25, 50, 100, 200, or 400 *μ*g/mL HET, JTT, or NYT (manufactured by Tsumura [TJ], Kracie [KR], and Kotaro [KO]). PBMC proliferation, CD4^+^ T cell, CD8^+^ T cell, and regulatory T cell (Treg) proportions and interleukin (IL) concentrations (IL-6, IL-10, IL-17A, interferon-*γ*, tumor necrosis factor-*α*, and transforming growth factor (TGF)-*β*) secreted by PBMCs were measured using Cell Counting Kit-8 or flow cytometry bead analysis. PBMC proliferation and CD4^+^ T cell percentages were similar in the HET, JTT, NYT, and control groups; however, the percentage of CD8^+^ T cells tended to increase after treatments. Tregs were suppressed by HET, JTT, and NYT, and TJ-JTT significantly decreased Treg numbers (compared with control). The concentrations of all cytokines except TGF-*β* were increased in a concentration-dependent manner (*p* < 0.05); particularly, KR-HET induced IL-6 secretion (compared with the control, TJ-HET, and KO-HET; 37-, 7-, and 17-fold, respectively; *p* < 0.05). The TGF-*β* concentration was decreased in a concentration-dependent manner by HET, JTT, and NYT (compared with the control). These results suggest that, compared with TJ-HET and KO-HET, KR-HET should be administered with caution. Although HET, JTT, and NYT belong to the same Hozai group and have the same names among companies, their differing effects on immune activity must be considered and they must be administered with caution.

## 1. Introduction

Hozai, a group of Japanese traditional herbal medicines (Kampo), has been used to treat patients with fatigue, including patients with cancer who are physically and mentally weakened [1, 2]. Hochuekkito (HET), Juzentaihoto (JTT), and Ninjin'yoeito (NYT) are representative of Hozai and are marketed by several companies in Japan; although their formulations contain different types and amounts of herbs, they have been given the same name by different companies (Table 1).

These medicines modulate or enhance the immune system [3]. Mori et al. [4] reported that HET inhibits influenza virus infection by enhancing host immune responses in mice. HET has also been shown to reduce cancer-related fatigue [5] and prevent chemically induced cancer [6]. JTT improves the function of natural killer cells and suppresses the immune inhibitory system, thereby preventing an excessive immune response in patients with general fatigue [7]. Additionally, JTT increases the regulatory activities of T cells by decreasing the forkhead box p3 (Foxp3)^+^ regulatory T cell (Treg) population in patients with advanced pancreatic cancer [8]. The symptoms of fatigue caused by nab-paclitaxel plus gemcitabine treatment administered to patients with unresectable pancreatic cancer have been reported to be improved by NYT [9]. Furthermore, NYT has been shown to synergistically enhance the effects of a tumor vaccine mediated by CD8^+^ T cells [10]. However, the immunological characteristics of HET, JTT, and NYT manufactured by different companies are unclear.

Moreover, physicians in Japan may not comprehensively understand these Kampo medicines and the differences between each company's Hozai. For many physicians trained mostly in Western medicine, understanding Kampo theories is difficult and requires an extensive amount of time and experience [11]. An Internet survey of Japanese physicians revealed concerns regarding Kampo's efficacy, safety, and cost and that the physicians commonly used or advised on Kampo medicines in clinical practice [12]. For physicians to prescribe an appropriate Kampo medicine to each patient, a good understanding of “*Sho* (pattern),” which consists of “*Qi*, *Blood*, and *Water*,” and the difference among different Hozai formulations (although all of them tonify “*Qi*”) is required [11]. Further studies can provide useful evidence for clinical professionals who find it difficult to use Kampo medicine.

In this study, we aimed to investigate the immunological differences in human peripheral blood mononuclear cells (PBMCs) treated with HET, JTT, and NYT manufactured by different pharmaceutical companies to provide evidence for their appropriate use in patients.

## 2. Materials and Methods

### 2.1. Materials and Drug Preparation

HET, JTT, and NYT were purchased from Tsumura & Co. (TJ; Tokyo, Japan), Kracie Pharma Ltd. (KR; Tokyo, Japan), and Kotaro Pharmaceutical Co., Ltd. (KO; Osaka, Japan) (Table 1). HET, JTT, and NYT (10 mg) were dissolved in 2 mL of Roswell Park Memorial Institute- (RPMI-) 1640 medium containing 10% fetal bovine serum (FBS), centrifuged at 2190xg for 10 min at 23°C, and then passed through a 0.22 *μ*m membrane filter (ADVANTEC MFS, Inc., Tokyo, Japan). The nine Hozai solutions were stored at −20°C until use. FBS and RPMI-1640 medium were purchased from Sigma-Aldrich (St. Louis, MO, USA). Penicillin-streptomycin (10,000 U/mL penicillin and 10,000 *μ*g/mL streptomycin) and 10x phosphate-buffered saline (PBS) were purchased from Gibco (Grand Island, NY, USA). Concanavalin A was purchased from Wako Pure Chemical Corporation (Osaka, Japan), and Cell Counting Kit-8 (CCK-8) was obtained from Dojindo Molecular Technologies (Kumamoto, Japan). Paraformaldehyde was purchased from Thermo Fisher Scientific (Waltham, MA, USA). PerCP-Cy™5.5 mouse anti-human CD4, allophycocyanin (APC) mouse anti-human CD8, APC mouse anti-human CD25, Human Foxp3 Buffer A, Human Foxp3 Buffer B, phycoerythrin (PE) mouse anti-human Foxp3, BD Cytometric Bead Assay (CBA) Flex Set, and Human TGF-*β* Single Plex Flex Set were purchased from BD Biosciences (Franklin Lakes, NJ, USA).

### 2.2. Isolation of PBMCs

Venous blood (20 mL) was collected from healthy subjects (*n* = 6, 1 male and 5 females; average age, 22.4 ± 0.53 years). Written informed consent to participate in the study was obtained from each participant. This study was approved by the Ethical Committee of the Tokyo University of Pharmacy and Life Sciences (approval no. 17–33).

The heparinized blood was loaded onto 4 mL of Ficoll-Hypaque (Nacalai Tesque, Kyoto, Japan) and centrifuged at 1300xg for 20 min to isolate the PBMCs. To evaluate the effects of Hozai on PBMCs, the cells were washed and resuspended in RPMI-1640 medium containing 10% FBS and penicillin-streptomycin at a final density of 1 × 10^6^ cells/mL.

### 2.3. PBMC Proliferation

We loaded PBMC suspension (186 *μ*L) into each well of a 96-well plate. Concanavalin A (10 *µ*L), a T cell mitogen, was added to each well at a final concentration of 5.0 *μ*g/mL; 4 *μ*L of each Hozai was added at final concentrations of 25, 50, 100, 200, and 400 *μ*g/mL. The concentration range was determined based on a previous study, in which the serum levels of several types of Kampo were estimated to be 200–300 *μ*g/mL after 1 week of administration [13]. Four microliters of medium containing 10% FBS was added to the control wells. The plate was incubated for 72 h at 37°C in a humidified atmosphere containing 5% CO_2_. Next, 10 *μ*L of CCK-8 reagent was added to each well according to the manufacturer's protocol, followed by an additional incubation for 3 h at 37°C. The optical density (OD) of sample in each well was measured at 450 nm. The proliferation of PBMC was calculated as follows: PBMC proliferation rate = (OD sample value−OD blank value)/(OD control value−OD blank value) × 100.

### 2.4. T Cell Subset Analysis

The PBMC suspension (744 *µ*L) was seeded into each well of a 24-well plate along with 40 *μ*L of concanavalin A to a final concentration of 5.0 *μ*g/mL. Subsequently, 16 *μ*L each of Hozai and medium (as control: 0 *μ*g/mL) was added to obtain the same final concentrations as those used in the PBMC proliferation assay. The plate was incubated for 72 h at 37°C in a humidified atmosphere containing 5% CO_2_. The culture supernatants were stored at −80°C before measuring the cytokine concentrations (see below). PBMCs were centrifuged at 302xg at 23°C for 5 min and then resuspended in PBS/1% FBS (wash buffer) before use in T cell subset analysis.

To evaluate CD4^+^ and CD8^+^ T cells, 10 *μ*L of PerCP-Cy™5.5 mouse anti-human CD4 and 5 *μ*L of APC mouse anti-human CD8 were added to the PBMC suspension and incubated at 37°C for 20 min in the dark. After washing the cells with washing buffer, 0.4 mL of staining buffer (PBS/1% FBS/0.4% paraformaldehyde) was added to the cell suspension, which was then passed through a 0.37 *μ*m membrane filter (ADVANTEC MFS) before flow cytometry.

To evaluate CD4^+^CD25^+^Foxp3^+^ Tregs, 10 *μ*L of PerCP-Cy™5.5 mouse anti-human CD4 and 10 *μ*L of APC mouse anti-human CD25 were added to the cell suspensions after incubation. The cell suspensions were incubated for 20 min at 37°C in the dark, after which the cells were washed, and 1 mL of Human Foxp3 Buffer A diluted 10-fold with distilled water was added to the cell suspension, which was then incubated for 10 min in the dark at 20–26°C. The cells were washed with washing buffer, resuspended in 0.2 mL of Human Foxp3 Buffer B diluted 50-fold with Human Foxp3 Buffer A, and incubated for 30 min in the dark at room temperature. After washing the cells, 10 *μ*L of PE mouse anti-human Foxp3 was added, and the cell suspension was incubated for 30 min at 37°C in the dark. After washing the PBMCs, 0.4 mL of staining buffer was added to the cell suspension and passed through a 0.37 *μ*m membrane filter before flow cytometry.

A total of 3 × 10^4^ cells were analyzed by flow cytometry (FACSCanto and FACSDiva software v6.0; BD Biosciences). PBMCs in the lymphocyte fraction were gated, and the percentages of CD4^+^ and CD8^+^ T cells were calculated. The percentage of Tregs, as CD4^+^CD25^+^Foxp3^+^ T cells, was measured using methods similar to those used to measure CD4^+^ T cells and CD8^+^ T cells.

### 2.5. Evaluation of Cytokines Using the CBA Assay

The concentrations of interferon-*γ* (IFN-*γ*), tumor necrosis factor-*α* (TNF-*α*), and interleukin- (IL-) 6, IL-10, and IL-17A in the PBMC culture supernatants were measured using a BD CBA Flex Kit according to the manufacturer's instructions. The Human TGF-*β* Single Plex Flex Set was used to detect transforming growth factor- (TGF-) *β*. Data were analyzed using FCAP Array software ver3.0 (BD Biosciences). This assay employs a mixture of beads with distinct fluorescence intensity characteristics to simultaneously detect multiple cytokines in supernatant samples. Each bead was coated with an antibody directed against a specific cytokine, and fluorescent signals were detected by flow cytometry. The kit performance was optimized for analyzing physiologically relevant concentrations of cytokines over a broad dynamic range.

### 2.6. Statistical Analysis

All experiments were repeated more than three times, and the data are presented as mean + standard deviation (SD). *p* < 0.05 was considered to indicate statistically significant results. One-way analysis of variance (ANOVA) followed by Dunnett's multiple comparisons test was used to analyze PBMC proliferation, T cell subsets, and cytokine concentrations after HET, JTT, and NYT treatments compared with those after control treatment (*p* < 0.05 is shown as ^*∗*^). Differences in PBMC proliferation, T cell subsets, and cytokine concentrations among groups treated with Hozai from TJ, KR, and KO were analyzed using the one-way ANOVA followed by Tukey's multiple comparisons test (*p* < 0.05 is shown as ^†^). All statistical analyses were performed using GraphPad Prism 8.0 (GraphPad, Inc., La Jolla, CA, USA).

## 3. Results

### 3.1. PBMC Proliferation

**Figure 1:**
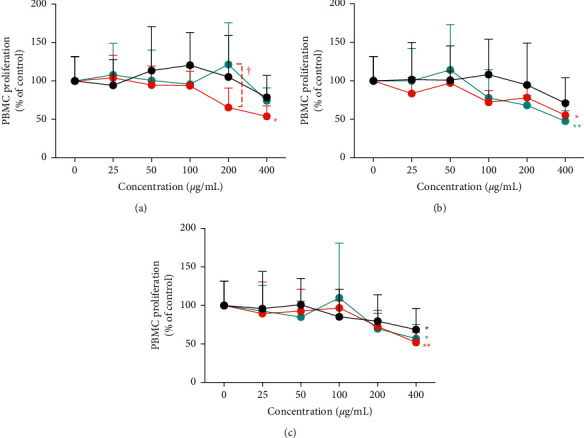
Proliferation of PBMCs treated with HET, JTT, or NYT cultured for 72 h at 37°C in a humidified atmosphere containing 5% CO_2_. Effects of (a) HET, (b) JTT, and (c) NYT on PBMC proliferation. Proliferation was evaluated using the CCK-8 assay after treatment with various concentrations (0–400 *μ*g/mL) of HET, JTT, or NYT manufactured by TJ (black), KR (red), or KO (cyan). Relative cell viability was calculated as the ratio of the absorbance at 450 nm of each treatment group to that of the corresponding untreated control group. Data are shown as mean + SD of more than three independent experiments and were analyzed using the two-way ANOVA followed by Dunnett's multiple comparisons test and Tukey's multiple comparisons test. ^*∗*^*p* < 0.05 or ^*∗∗*^*p* < 0.01 vs. control (0 *μ*g/mL) group, and ^†^*p* < 0.05 vs. the same concentration group among TJ, KR, and KO. HET, Hochuekkito; JTT, Juzentaihoto; NYT, Ninjin'yoeito; TJ, Tsumura; KR, Kracie; KO, Kotaro.

The proliferation of PBMCs treated with HET, JTT, and NYT manufactured by TJ, KR, and KO was determined using the CCK-8 assay. Cell proliferation tended to decrease after HET, JTT, and NYT treatments in a dose-dependent manner (Figure 1). The proliferation of cells treated with KR-HET (400 *μ*g/mL) decreased significantly compared with that of cells treated with the control (^*∗*^*p*=0.0184, Figure 1(a)). PBMC proliferation after treatment with 200 *μ*g/mL KR-HET was significantly lower than that after treatment with 200 *μ*g/mL KO-HET (†*p*=0.0181, Figure 1(a)). Although 400 *μ*g/mL KR-JTT and KO-JTT significantly decreased PBMC proliferation compared with the control (^*∗*^*p*=0.0231 and ^*∗∗*^*p*=0.0052, respectively; Figure 1(b)), there were no significant differences among groups treated with different concentrations of Hozai from TJ, KR, and KO (Figure 1(b)). Treatment with 400 *μ*g/mL TJ-, KR-, and KO-NYT significantly suppressed cell proliferation compared with the control; however, the effects of TJ-, KR-, and KO-NYT treatments were similar (^*∗*^*p*=0.0468, ^*∗∗*^*p*=0.0070, and ^*∗*^*p*=0.0193, respectively.  Figure 1(c)).

### 3.2. T Cell Subset Analysis

**Figure 2:**
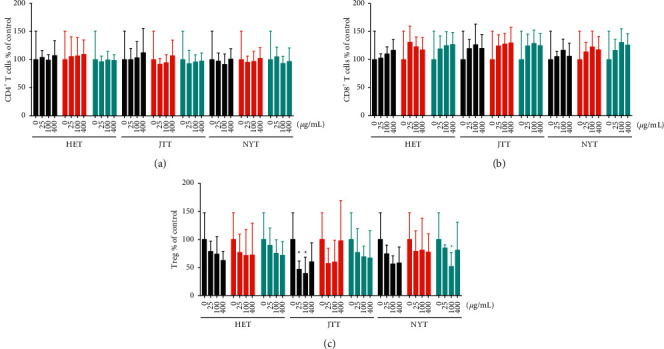
Effects of HET, JTT, and NYT on the percentage of CD4^+^ T cells, CD8^+^ T cells, and Tregs (CD4^+^CD25^+^Foxp3^+^ T cells). After treatment with different concentrations of HET, JTT, or NYT manufactured by TJ (black), KR (red), and KO (cyan), PBMCs were stained with PerCP-Cy™ 5.5 mouse anti-human CD4, APC mouse anti-human CD8, APC mouse anti-human CD25, and PE mouse anti-human Foxp3 to identify (a) CD4^+^ T cells, (b) CD8^+^ T cells, and (c) Tregs. Data are shown as mean + SD of more than three independent experiments. ^*∗*^*p* < 0.05 vs. control (0 *μ*g/mL) analyzed using the two-way ANOVA and Dunnett's multiple comparisons test. HET, Hochuekkito; JTT, Juzentaihoto; NYT, Ninjin'yoeito; TJ, Tsumura; KR, Kracie; KO, Kotaro.

The changes in the percentage of CD4^+^ and CD8^+^ cells and Tregs were determined by flow cytometry. HET, JTT, and NYT did not increase or decrease the percentage of CD4^+^ T cells compared with the control, including among groups treated with different concentrations of Hozai from TJ, KR, and KO (Figure 2(a) and Supplementary Figures S1–S3). In contrast, the percentage of CD8^+^ T cells tended to be increased by HET, JTT, or NYT treatment in a dose-dependent manner (Figure 2(b) and Supplementary Figures S4–S6). However, there were no significant differences among PBMCs treated with HET, JTT, and NYT from JT, KR, and KO (Figure 2(b)). Tregs were suppressed by HET, JTT, and NYT in a dose-dependent manner (Figure 2(c) and Supplementary Figures S7–S9). Treatment with 25 and 100 *μ*g/mL TJ-JTT and 100 *μ*g/mL KO-NYT significantly decreased the percentage of Tregs by 50% compared with the control (^*∗*^*p*=0.0413, 0.0167, and 0.0467, respectively, Figure 2(c)). TJ-JTT treatment reduced the percentage of Tregs more strongly than KR- or KO-JTT, HET, and NYT (Figure 2(c)).

### 3.3. Cytokine Analysis Using the CBA Assay

**Figure 3:**
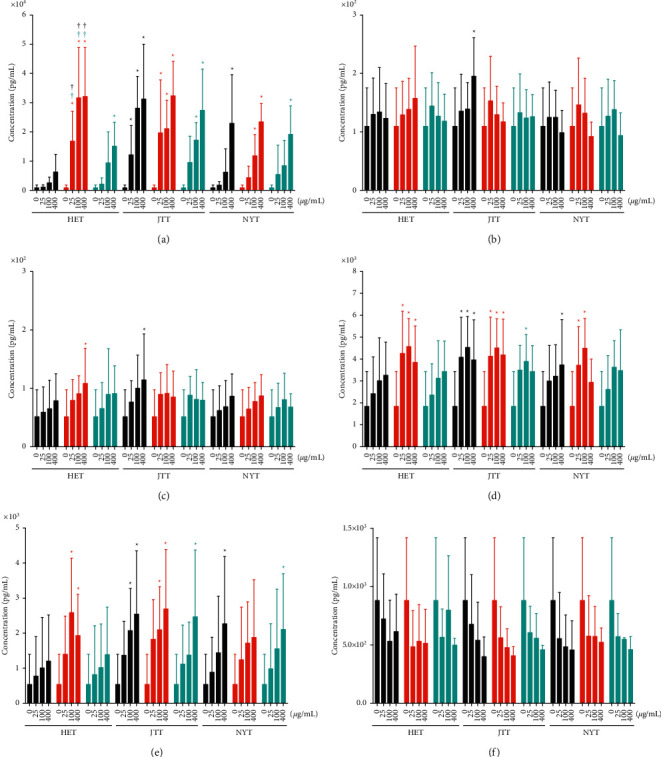
Cytokine concentrations in the supernatant of PBMCs treated with 0–400 *μ*g/mL HET, JTT, or NYT manufactured by TJ (black), KR (red), and KO (cyan). After 72 h of culture, the supernatant was analyzed to determine the (a) IL-6, (b) IL-10, (c) IL-17A, (d) IFN-*γ*, (e) TNF-*α*, and (f) TGF-*β* concentrations using bead-array followed by flow cytometry, as described in the Materials and Methods. Data are shown as mean + SD of more than three independent experiments; they were analyzed using two-way ANOVA followed by Dunnett's multiple comparisons test and Tukey's multiple comparisons test. ^*∗*^*p* < 0.05 vs. control (0 *μ*g/mL) group, and ^†^*p* < 0.05 vs. the same concentration group among TJ, KR, and KO. HET, Hochuekkito; JTT, Juzentaihoto; NYT, Ninjin'yoeito; TJ, Tsumura; KR, Kracie; KO, Kotaro.

IL-6 secretion was significantly increased compared with the control by 400 *μ*g/mL HET, JTT, and NYT in a dose-dependent manner (Figure 3(a)). There was no significant difference between groups treated with the same concentrations of JTT and NYT from TJ, KR, and KO; however, JTT tended to promote the secretion of IL-6, even at low concentrations, compared with NYT. In contrast, KR-HET significantly promoted the secretion of IL-6 compared with TJ-HET and KO-HET. Significantly increased secretion of IL-6 from PBMCs was observed following treatment with 25, 100, and 400 *μ*g/mL KR-HET compared with treatment with the same concentrations of TJ-HET and KO-HET (^†^*p* < 0.05). The IL-6 concentration in the supernatant of PBMCs treated with 400 *μ*g/mL TJ- and KO-HET was approximately 7-fold (6293.3 pg/mL) and 17-fold (15,087.2 pg/mL) higher than that of the control (868.3 pg/mL); that of PBMCs treated with 400 *μ*g/mL KR-HET was approximately 37-fold (32,126.9 pg/mL) higher than that of the control (^*∗*^*p*=0.0001). IL-10 was not significantly affected by HET, JTT, and NYT compared with the control, except upon treatment with 400 *μ*g/mL TJ-JTT, and there were no significant differences among groups treated with HET, JTT, and NYT (Figure 3(b)). HET, JTT, and NYT tended to promote the secretion of IL-17A in a dose-dependent manner, and 400 *μ*g/mL KR-HET and TJ-JTT significantly increased the concentration of IL-17 compared with the control. However, there was no significant difference among groups treated with HET, JTT, and NYT (Figure 3(c)). The IFN-*γ* concentration was increased by HET, JTT, and NYT in a dose-dependent manner, and 25 *μ*g/mL KR-HET, TJ-JTT, and KR-JTT significantly promoted the secretion of IFN-*γ* compared with the other treatments (Figure 3(d)). KR-HET increased the IFN-*γ* concentration more strongly than TJ-HET and KO-HET. Similar to IL-6 and IFN-*γ*, the TNF-*α* concentration was dose-dependently increased by treatment with HET, JTT, and NYT (Figure 3(e)). The cells treated with 100 *μ*g/mL KR-HET, TJ-JTT, and KR-JTT and 400 *μ*g/mL KR-HET, TJ-JTT, KR-JTT, KO-JTT, TJ-NYT, and KO-NYT showed significantly increased TNF-*α* production compared with the control. Only KR-HET significantly induced the production of TNF-*α* compared with TJ-HET and KO-HET. Among the cytokines, only the TGF-*β* concentration was decreased by HET, JTT, and NYT at all concentrations (compared with the control), and Hozai from TJ, KR, and KO suppressed the secretion of TGF-*β* to similar degrees (Figure 3(f)).

## 4. Discussion

We compared the immunological differences in PBMCs between groups of cells treated with HET, JTT, and NYT manufactured by the pharmaceutical industries TJ, KR, and KO to provide evidence for their appropriate use in patients. The results showed that KR-HET significantly suppressed PBMC proliferation even at a low dose (200 *μ*g/mL) compared with TJ- and KO-HET, JTT, and NYT and induced the secretion of IL-6 more significantly than TJ-HET and KO-HET. Additionally, only TJ-JTT significantly decreased the percentage of Tregs.

Previous studies have shown that Hozai is effective for treating fatigue because of its immune-stimulatory effects [8, 9, 14, 15]. However, no study has focused on the differences among HET, JTT, and NYT or those among these agents from different pharmaceutical industries in Japan, including their immunological effects on PBMCs. Generally, Hozai contains the following five herbal medicines: ginseng, *Astragalus* root, Japanese *Angelica* root, *Glycyrrhiza*, and *Atractylodes* rhizome or *Atractylodes lancea* rhizome. Kampo formulations for prescriptions in Japan contain different components from *Atractylodes* species; those marketed by some pharmaceutical companies contain byakujutsu or *Atractylodes* rhizome, whereas others contain sojutsu or *A. lancea* rhizome, although the name of the Kampo formulation is the same [16]. Additionally, although the amounts of herbal medicines are the same in KR-HET and KO-HET, the total amount extracted differs. As shown in Table 1, the medicines named HET, JTT, and NYT contain different components depending on the pharmaceutical company. However, many physicians do not understand these differences, as mastering them requires an extensive amount of time and experience [11]. Moreover, 70–90% of Japanese physicians regularly prescribe Kampo medicines according to clinical evidence [11]. Thus, it is necessary to determine the differences in the effects among HET, JTT, and NYT to ensure their safe and effective use in patients. Interestingly, here, there was no significant difference in PBMC proliferation after treatment with HET, JTT, and NYT manufactured by different companies, except for 200 *μ*g/mL KR-HET compared with 200 *μ*g/mL TJ-HET and KO-HET (Figure 1(a)). Furthermore, HET, JTT, and NYT did not change the percentage of CD4^+^ T cells (Figure 2(a)). However, the percentage of CD8^+^ T cells tended to increase following treatment with HET, JTT, or NYT in a dose-dependent manner (Figure 2(b)), whereas Tregs were suppressed by HET, JTT, and NYT (Figure 2(c)). Particularly, TJ-JTT significantly decreased the Treg percentage compared with the control, whereas KR-JTT and KO-JTT did not exert this effect. This finding suggests that it is not appropriate to administer these Kampo medicines (*Qi*-tonifying formulae) to patients with collagen disease without considering “*Sho*” because the aim of the disease treatment is to suppress immune abnormality.

The pharmacological effects of JTT on the immune and hematopoietic systems have been examined previously, and several types of active pectic polysaccharides have been isolated as active ingredients with immune-modulating activities, such as the lignin-carbohydrate complex-containing fraction obtained from TJ-JTT [17]. The oral administration of JTT increases and prolongs antibody production, particularly following influenza vaccination [18]. Furthermore, JTT has been shown to exert immune adjuvant effects on tumor vaccine therapy in a murine tumor model [19]. These observations strongly support that JTT promotes immunity. Moreover, here, only TJ-JTT significantly reduced the percentage of Tregs (Figure 2(c)), which are well-known immune suppressors [20]. The greatest difference in the composition of Hozai between each JTT is the inclusion of *A. lancea* rhizome, sojutsu or *Atractylodes* rhizome, or byakujutsu (Table 1). Some studies have compared these crude drugs but did not evaluate the Treg counts. Our results suggest that sojutsu in JTT suppresses Tregs more strongly than byakujutsu.

HET and NYT also exert immune effects. The enhancement of granulocyte colony-stimulating factor secretion induced by HET may partially contribute to the clinically observed pharmacological activities of HET, including its immunomodulating activity [21]. NYT synergistically enhances whole-tumor cell vaccine effects *in vivo*, which requires CD8^+^ T cells [10]. In addition, reduced numbers of Tregs are more apparent in vaccinated mice fed a diet containing NYT than in those fed a control diet [10]. Our results show that Hozai slightly increased the number of CD8^+^ T cells and enhanced the immune response, with no significant differences observed among the Hozai formulations.

KR-HET suppressed PBMCs more strongly than TJ-HET and KO-HET and considerably promoted IL-6 secretion compared with TJ-HET and KO-HET. The IL-6 concentration in the supernatant of PBMCs treated with 400 *μ*g/mL TJ- and KO-HET was approximately 7- and 17-fold higher than that in the supernatant of PBMCs treated with the control, whereas that of PBMCs treated with 400 *μ*g/mL KR-HET was approximately 37-fold higher than that of cells treated with the control. JTT also increased the IL-6 concentration more than HET and NYT, regardless of the source company. IL-6 is a well-known inflammatory cytokine that promotes cancer growth and metastasis [22]. Therefore, our results suggest that the administration of KR-HET and JTT should be considered for patients with cancer, rheumatoid arthritis, inflammatory bowel disease, and Castleman's disease; however, they might be effective in preventing infectious diseases in weak patients as a merit. In contrast, 400 *μ*g/mL TJ-HET did not significantly increase the IL-6 concentration, similar to HET, JTT, and NYT; therefore, JT-HET appears to be safer because it does not induce the secretion of IL-6.

Although the concentration of IL-6 was substantially increased by Hozai when compared with the control, that of IL-10, IL-17A, IFN-*γ*, and TNF-*α* was only moderately increased in a dose-dependent manner (Figures 3(b)–3(e)). In contrast, the concentration of TGF-*β* was decreased when compared with the control by HET, JTT, and NYT (Figure 3(f)). Treatment with >25 *μ*g/mL Hozai tended to show the same inhibitory effects, and the concentration of TGF-*β* secreted from PBMCs treated with 25 and 400 *μ*g/mL Hozai was approximately the same. T cells secrete different cytokines depending on the T cell subtype. For example, T helper 1 (Th1) cells, which evoke cell-mediated immunity and phagocyte-dependent inflammation, produce IFN-*γ*, IL-2, and TNF. Th2 cells, which evoke strong antibody responses and eosinophil accumulation, produce IL-4, IL-5, IL-6, IL-9, IL-10, and IL-13 [23]. Our results suggest that HET, JTT, and NYT did not greatly increase the percentage of CD4^+^ and CD8^+^ T cells but increased immune activity by enhancing IL-6, IL-10, IL-17A, IFN-*γ*, and TNF-*α* secretion. In contrast, HET, JTT, and NYT decreased the percentage of Tregs and, accordingly, TGF-*β* secretion.

In cancer palliative medicine, HET or JTT tend to be prescribed initially; in serious cases, they are replaced with NYT [24]. Our results suggest that HET, JTT, and NYT should be administered to patients based on their physical status while considering the composition differences among formulations from different pharmaceutical companies, as inappropriate prescription may worsen the patient's condition. Our findings provide supportive evidence for the appropriate use of Hozai.

A limitation of this study is that we used human PBMCs activated with concanavalin A and evaluated the cells *in vitro*. Thus, further studies are required to determine the differences among HET, JTT, and NYT in terms of their absorption, distribution, metabolism, and excretion *in vivo*. Additionally, the chemical composition of the formulations must be explored and the main chemical components should be identified and quantified in further study.

## 5. Conclusions

In summary, we found that low doses of KR-HET suppressed PBMC proliferation and induced IL-6 secretion more significantly than TJ-HET and KO-HET. Additionally, only TJ-JTT significantly decreased the percentage of Tregs. These results suggest that the administration of KR-HET should be considered and that the IL-6 concentration in patients receiving KR-HET should be monitored. TJ-JTT may increase immunoactivity via Treg suppression more than the other JTT formulations evaluated. HET, JTT, and NYT belong to the same Hozai group and have the same names among different companies but showed different immune activities. Thus, these immunological differences should be considered with caution while administering HET, JTT, and NYT to patients.

## Figures and Tables

**Table 1:** Composition of HET, JTT, and NYT extract granules manufactured by Tsumura, Kracie, and Kotaro.

	Volume (g)								
	HET			JTT			NYT		
	TJ^(a)^	KR^(b)^	KO^(c)^	TJ^(a)^	KR^(d)^	KO^(e)^	TJ^(f)^	KR^(g)^	KO^(h)^
JP Ginseng	4.0	4.0	4.0	3.0	3.0	2.5	3.0	3.0	3.0
JP *Atractylodes lancea* rhizome	4.0	—	—	3.0	—	—	—	—	—
JP *Atractylodes* rhizome	—	4.0	4.0	—	3.0	3.5	4.0	4.0	4.0
JP *Astragalus* root	4.0	4.0	4.0	3.0	3.0	2.5	1.5	1.5	1.5
JP Japanese *Angelica* root	3.0	3.0	3.0	3.0	3.0	3.5	4.0	4.0	4.0
JP *Glycyrrhiza*	1.5	1.5	1.5	1.5	1.5	1.0	1.0	1.0	1.0
JP *Bupleurum* root	2.0	2.0	2.0	—	—	—	—	—	—
JP *Cimicifuga* rhizome	1.0	1.0	1.0	—	—	—	—	—	—
JP Jujube	2.0	2.0	2.0	—	—	—	—	—	—
JP *Citrus* Unshiu peel	2.0	2.0	2.0	—	—	—	2.0	2.0	2.0
JP Ginger	0.5	0.5	0.5	—	—	—	—	—	—
JP Poria *Sclerotium*	—	—	—	3.0	3.0	3.5	4.0	4.0	4.0
JP *Rehmannia* root	—	—	—	3.0	3.0	3.5	4.0	4.0	4.0
JP *Cnidium* rhizome	—	—	—	3.0	3.0	3.0	—	—	—
JP Peony root	—	—	—	3.0	3.0	3.0	2.0	2.0	2.0
JP Cinnamon bark	—	—	—	3.0	3.0	3.0	2.5	2.5	2.5
JP *Polygala* root	—	—	—	—	—	—	2.0	2.0	2.0
JP *Schisandra* fruit	—	—	—	—	—	—	1.0	1.0	1.0

^a)^7.5 g of each extract fine granule contains 5.0 g of dried extract of the above mixed crude drugs. ^b)^7.5 g contains 6.4 g of dried extract of the above mixed crude drugs. ^c)^12.0 g of each extract fine granules contains 7.0 g of dried extract of the above mixed crude drugs. ^d)^7.5 g contains 6.2 g of dried extract of the above mixed crude drugs. ^e)^15.0 g of each fine granule extract contains 8.5 g of dried extract of the above mixed crude drugs. ^f)^9.0 g of each fine granule extract contains 6.0 g of dried extract of the above mixed crude drugs. ^g)^7.5 g of each fine granule extract contains 6.7 g of dried extract of the above mixed crude drugs. ^h)^15.0 g of each extract fine granule contains 9.2 g of dried extract of the above mixed crude drugs. JP, Japanese Pharmacopeia; HET, Hochuekkito; JTT, Juzentaihoto; NYT, Ninjin'yoeito; TJ, Tsumura; KR, Kracie; KO, Kotaro.

## Data Availability

The data supporting the findings of this study are available from the corresponding author upon request.

## Supplementary Materials

Supplementary Materials The following supplementary information is available for this paper: Supplementary Figure S1: flow cytometry plots and histograms of CD4^+^ T cells treated with HET. Supplementary Figure S2: flow cytometry plots and histograms of CD4^+^ T cells treated with JTT. Supplementary Figure S3: flow cytometry plots and histograms of CD4^+^ T cells treated with NYT. Supplementary Figure S4: flow cytometry plots and histograms of CD8^+^ T cells treated with HET. Supplementary Figure S5: flow cytometry plots and histograms of CD8^+^ T cells treated with JTT. Supplementary Figure S6: flow cytometry plots and histograms of CD8^+^ T cells treated with NYT. Supplementary Figure S7: flow cytometry plots and histograms of Tregs treated with HET. Supplementary Figure S8: flow cytometry plots and histograms of Tregs treated with JTT. Supplementary Figure S9: flow cytometry plots and histograms of Tregs treated with NYT.
