# Supplementary material for: Immunological Differences in Human Peripheral Blood Mononuclear Cells Treated with Traditional Japanese Herbal Medicines Hochuekkito, Juzentaihoto, and Ninjin'yoeito from Different Pharmaceutical Companies
Source: Evid Based Complement Alternat Med. 2021 Sep 18;2021:7605057. doi: 10.1155/2021/7605057 (PMC8476247; doi:10.1155/2021/7605057)
Supplement: Supplementary Materials — The following supplementary information is available for this paper: Supplementary Figure S1: flow cytometry plots and histograms of CD4+ T cells treated with HET. Supplementary Figure S2: flow cytometry plots and histograms of CD4+ T cells treated with JTT. Supplementary Figure S3: flow cytometry plots and histograms of CD4+ T cells treated with NYT. Supplementary Figure S4: flow cytometry plots and histograms of CD8+ T cells treated with HET. Supplementary Figure S5: flow cytometry plots and histograms of CD8+ T cells treated with JTT. Supplementary Figure S6: flow cytometry plots and histograms of CD8+ T cells treated with NYT. Supplementary Figure S7: flow cytometry plots and histograms of Tregs treated with HET. Supplementary Figure S8: flow cytometry plots and histograms of Tregs treated with JTT. Supplementary Figure S9: flow cytometry plots and histograms of Tregs treated with NYT. [file 7605057.f1.pdf]

Supplementary Material

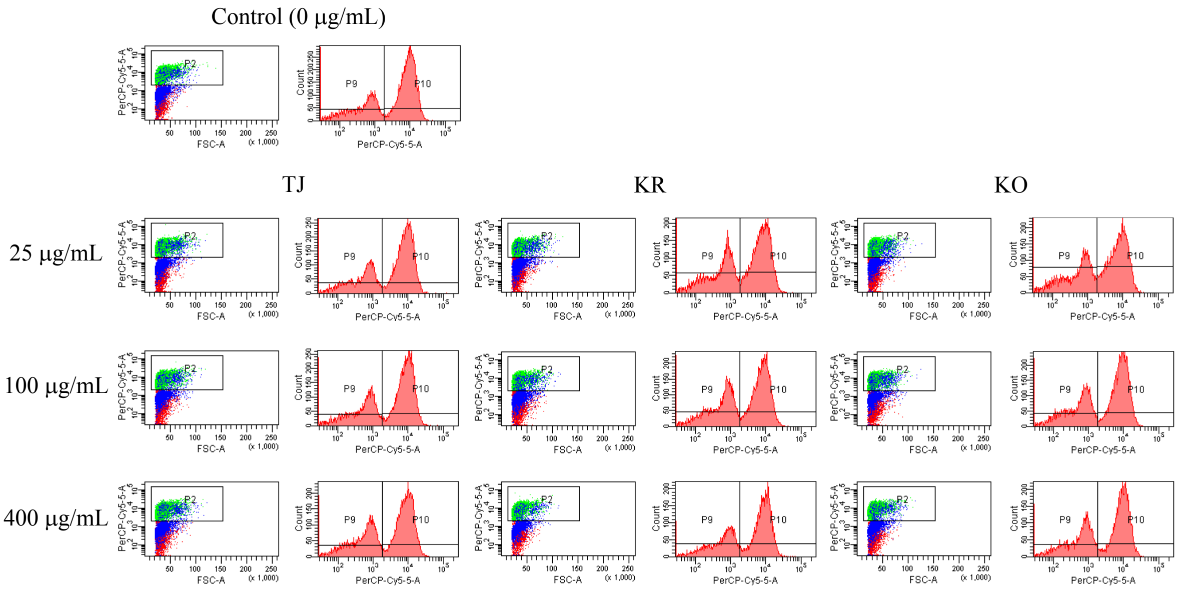

Figure S1. Flow cytometry plots and histograms of CD4$^+$ T cells treated with HET. PBMCs were treated with 0 (as control), 25, 100, or 400 μg/mL HET manufactured by TJ, KR, and KO and stained with PerCP-Cy^{TM}5.5 mouse anti-human CD4, and the lymphocyte fraction of PBMCs was gated for CD4$^+$ T cells. HET, Hochuekkito; TJ, Tsumura; KR, Kracie; KO, Kotaro.

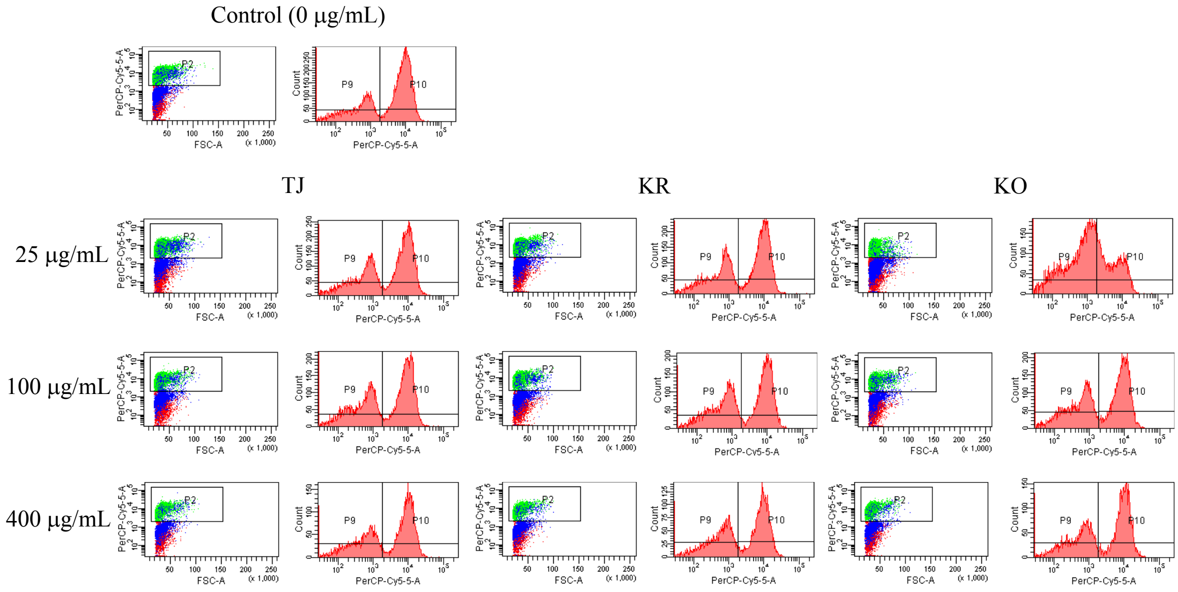

Figure S2. Flow cytometry plots and histograms of CD4$^+$ T cell treated with JTT. PBMCs were treated with 0 (as control), 25, 100, or 400 μg/mL JTT manufactured by TJ, KR, and KO and stained with PerCP-

Cy^{TM}5.5 mouse anti-human CD4, and the lymphocyte fraction of PBMCs was gated for CD4^{+} T cells. JTT, Juzentaihoto; TJ, Tsumura; KR, Kracie; KO, Kotaro.

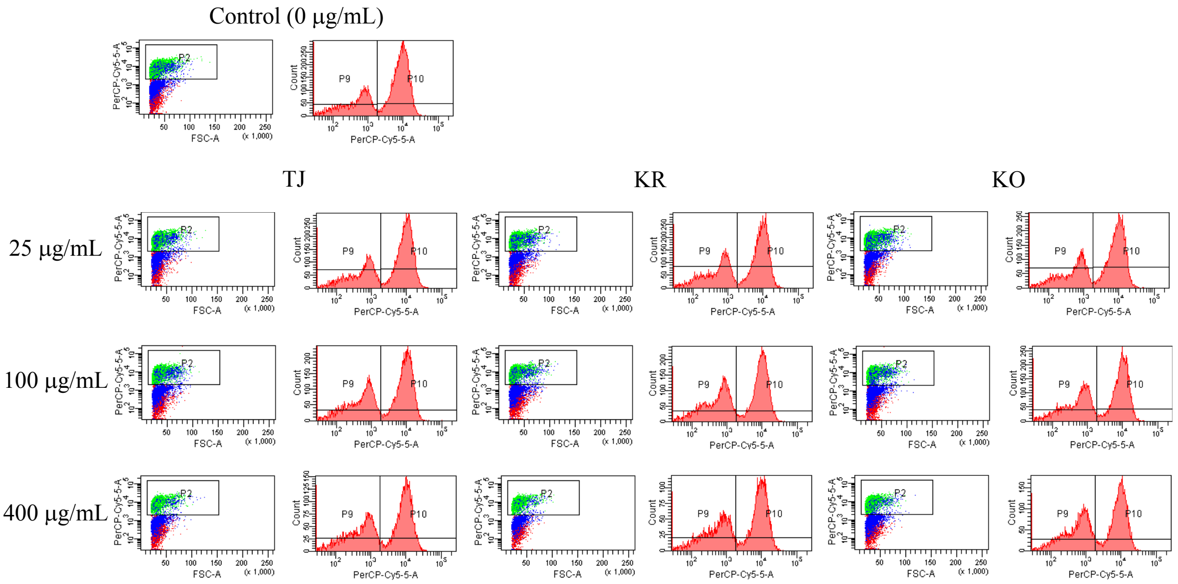

Figure S3. Flow cytometry plots and histograms of CD4^{+} T cells treated with NYT. PBMCs were treated with 0 (as control), 25, 100, or 400 μg/mL NYT manufactured by TJ, KR, and KO and stained with PerCP-Cy^{TM}5.5 mouse anti-human CD4, and the lymphocyte fraction of PBMCs was gated for CD4^{+} T cells. NYT, Ninjin'yoeito; TJ, Tsumura; KR, Kracie; KO, Kotaro.

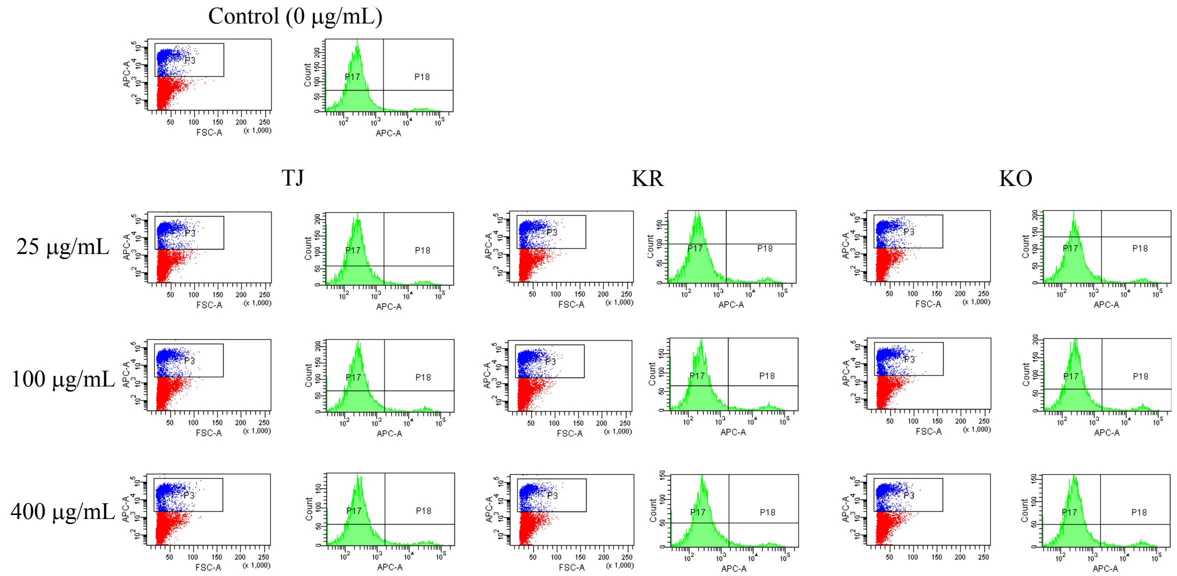

Figure S4. Flow cytometry plots and histograms of CD8^{+} T cells treated with HET. PBMCs were treated with 0 (as control), 25, 100, or 400 μg/mL HET manufactured by TJ, KR, and KO and stained with APC

mouse anti-human CD8, and the lymphocyte fraction of PBMCs was gated for CD8$^+$ T cells. HET, Hochuekkito; TJ, Tsumura; KR, Kracie; KO, Kotaro.

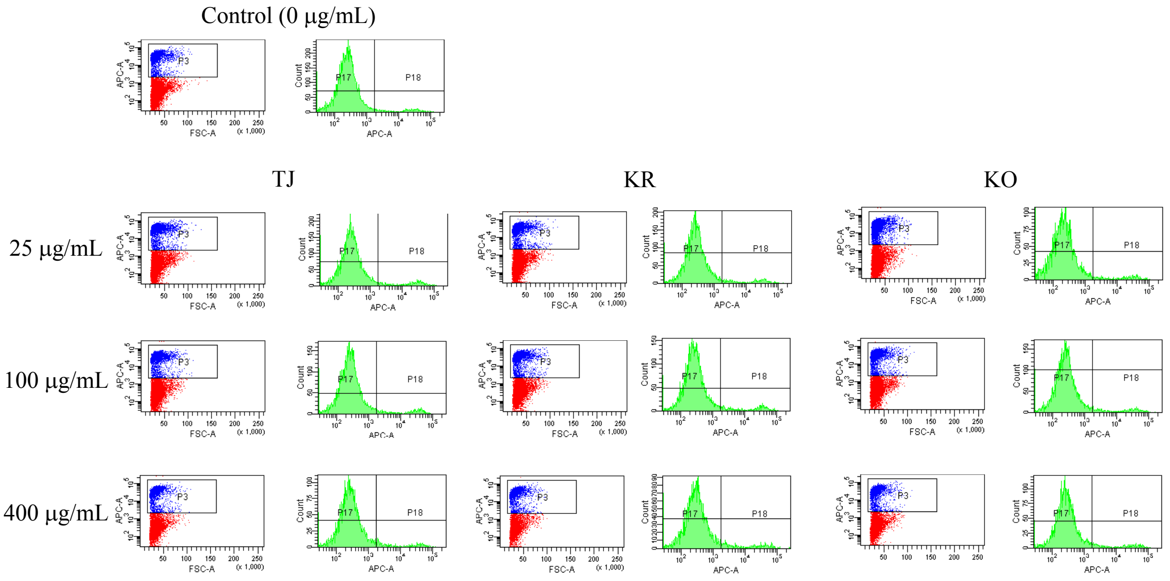

Figure S5. Flow cytometry plots and histograms of CD8$^+$ T cells treated with JTT. PBMCs were treated with 0 (as control), 25, 100, or 400 µg/mL JTT manufactured by TJ, KR, and KO and stained with APC mouse anti-human CD8, and the lymphocyte fraction of PBMCs was gated for CD8$^+$ T cells. JTT, Juzentaihoto; TJ, Tsumura; KR, Kracie; KO, Kotaro.

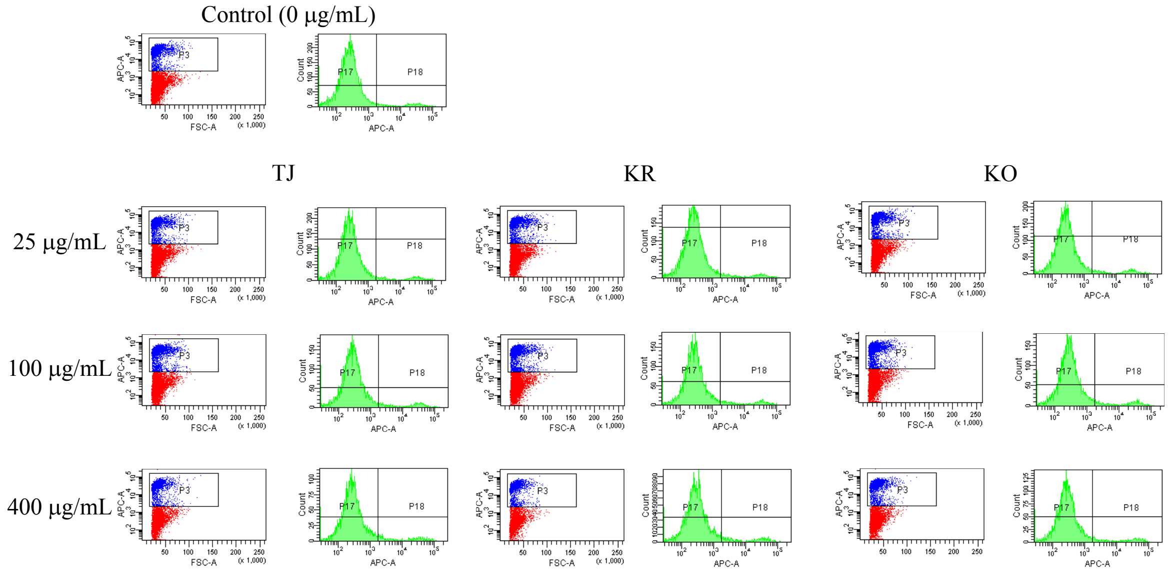

Figure S6. Flow cytometry plots and histograms of CD8$^+$ T cells treated with NYT. PBMCs were treated with 0 (as control), 25, 100, or 400 µg/mL NYT manufactured by TJ, KR, and KO and stained with APC

mouse anti-human CD8, and the lymphocyte fraction of PBMCs was gated for CD8$^+$ T cells. NYT, Ninjin'yoeito; TJ, Tsumura; KR, Kracie; KO, Kotaro.

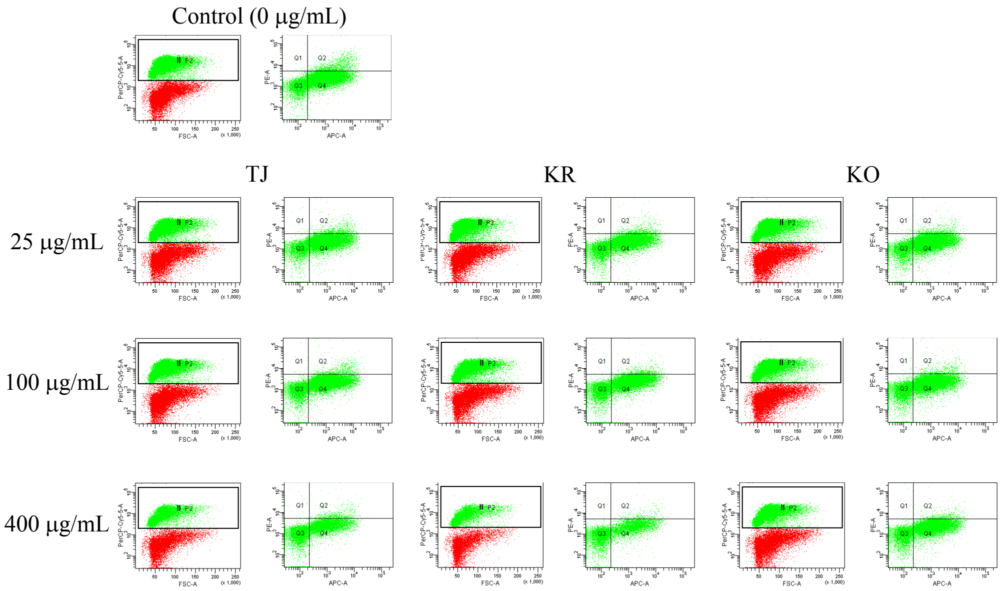

Figure S7. Flow cytometry plots and histograms of Tregs treated with HET. PBMCs were treated with 0 (as control), 25, 100, or 400 μg/mL HET manufactured by TJ, KR, and KO and stained with PerCP-Cy^{TM}5.5 mouse anti-human CD4, APC mouse anti-human CD25, and PE mouse anti-human Foxp3 to identify Treg. The lymphocyte fraction of PBMCs was gated for CD4$^+$ cells, and the percentages of CD25$^+$ (abscissa)/Foxp3$^+$ (ordinate)-double-positive cells in the CD4$^+$ cell fraction were calculated as Tregs. HET, Hochuekkito; TJ, Tsumura; KR, Kracie; KO, Kotaro.

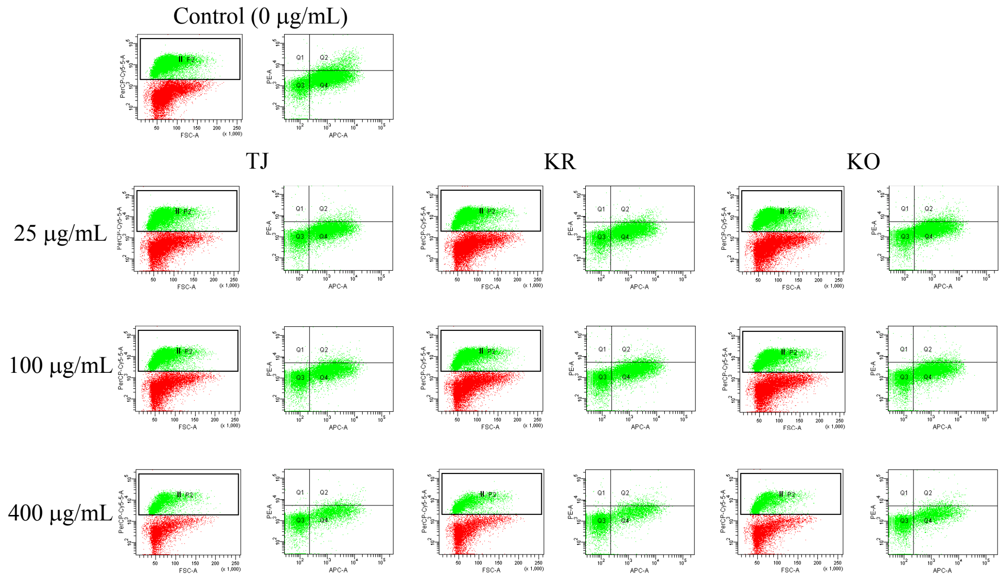

Figure S8. Flow cytometry plots and histograms of Tregs treated with JTT. PBMCs were treated with 0 (as control), 25, 100, or 400 µg/mL JTT manufactured by TJ, KR, and KO and stained with PerCP-Cy^{TM}5.5 mouse anti-human CD4, APC mouse anti-human CD25, and PE mouse anti-human Foxp3 to identify Tregs. The lymphocyte fraction of PBMCs was gated for CD4^{+} cells, and the percentages of CD25^{+} (abscissa)/Foxp3^{+} (ordinate)-double-positive cells in the CD4^{+} cell fraction were calculated as Tregs. JTT, Juzentaihoto; TJ, Tsumura; KR, Kracie; KO, Kotaro.

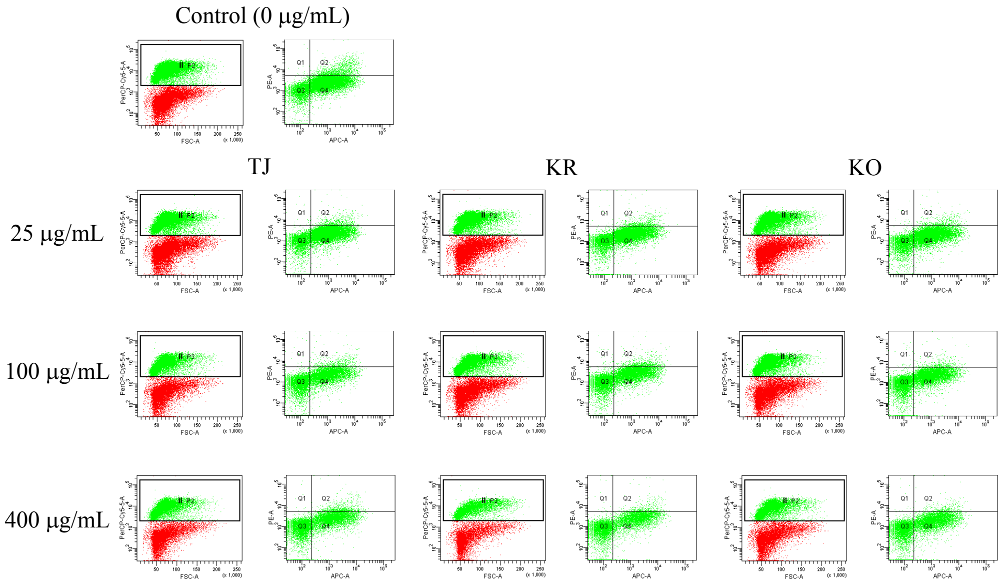

Figure S9. Flow cytometry plots and histograms of Tregs treated with NYT. PBMCs were treated with 0 (as control), 25, 100, or 400 µg/mL NYT manufactured by TJ, KR, and KO and stained with PerCP-Cy^{TM}5.5 mouse anti-human CD4, APC mouse anti-human CD25, and PE mouse anti-human Foxp3 to identify Tregs. The lymphocyte fraction of PBMCs was gated for CD4^{+} cells, and the percentages of CD25^{+} (abscissa)/Foxp3^{+} (ordinate)-double-positive cells in the CD4^{+} cell fraction were calculated as Tregs. NYT, Ninjin'yoeito; TJ, Tsumura; KR, Kracie; KO, Kotaro.
